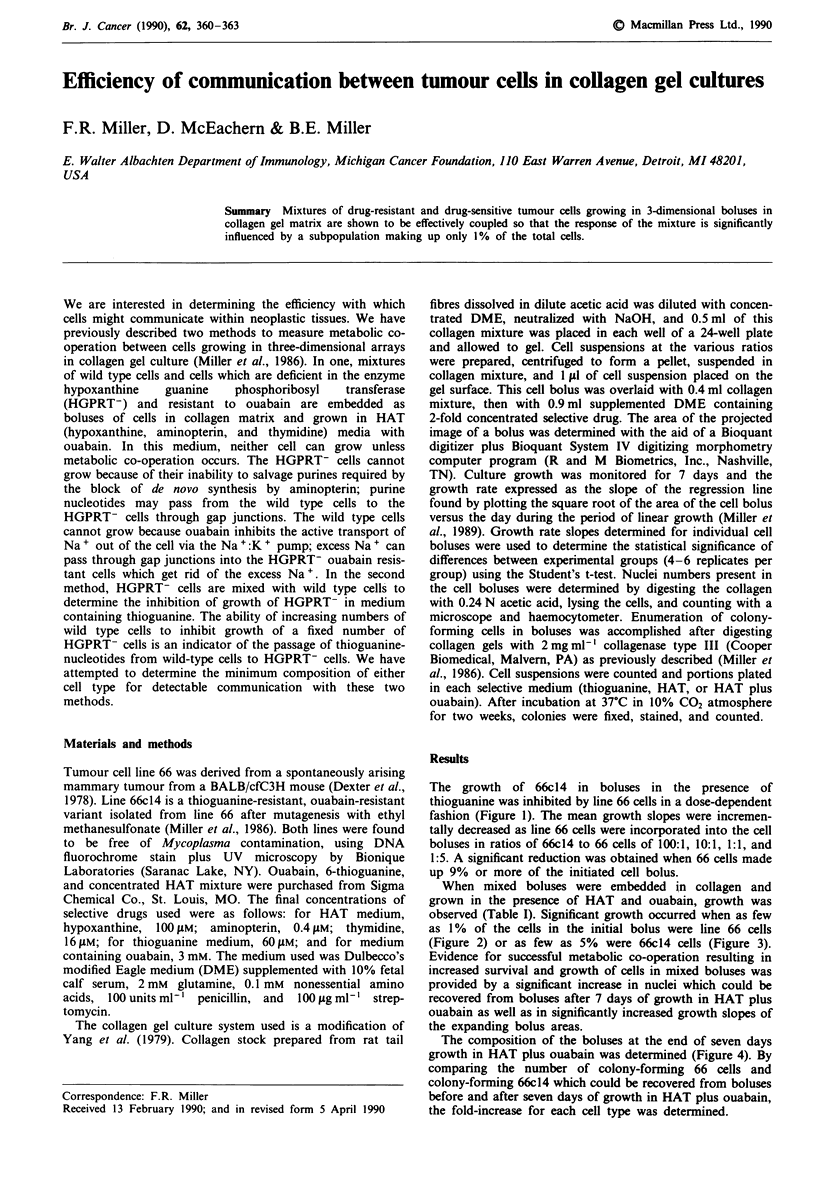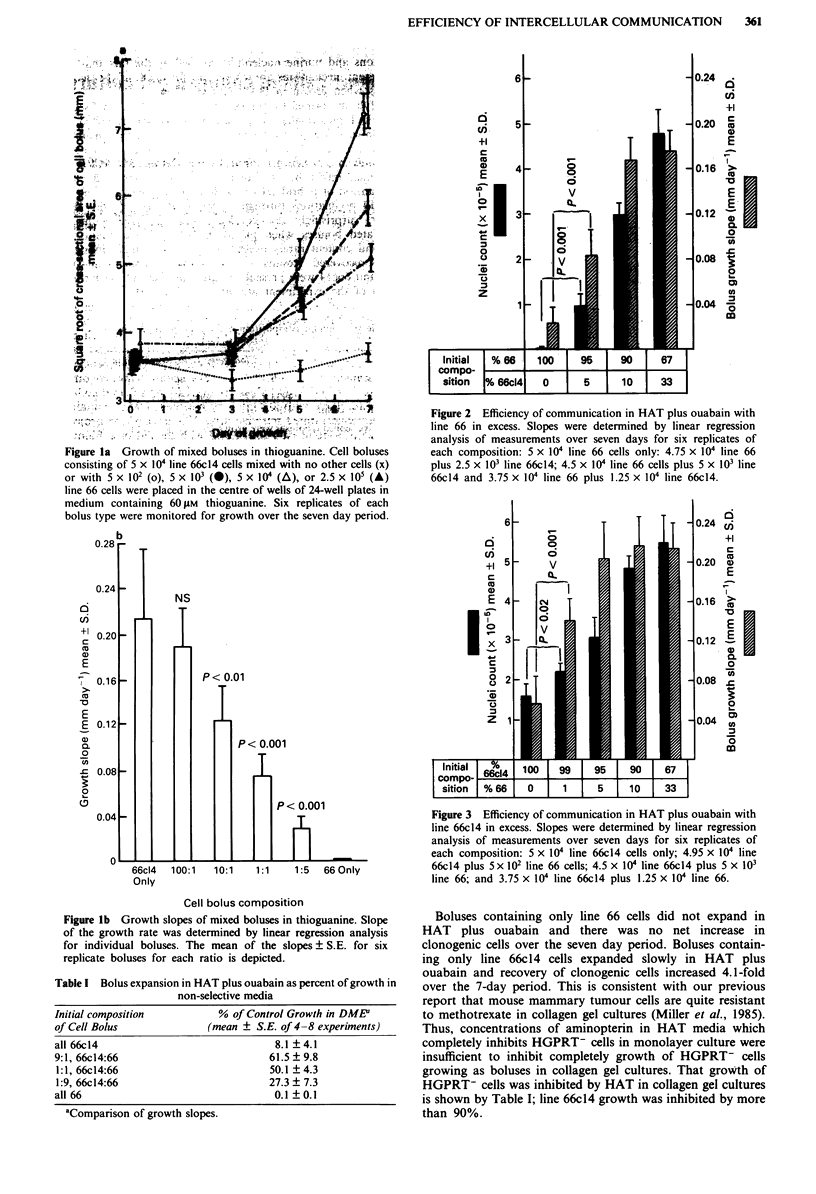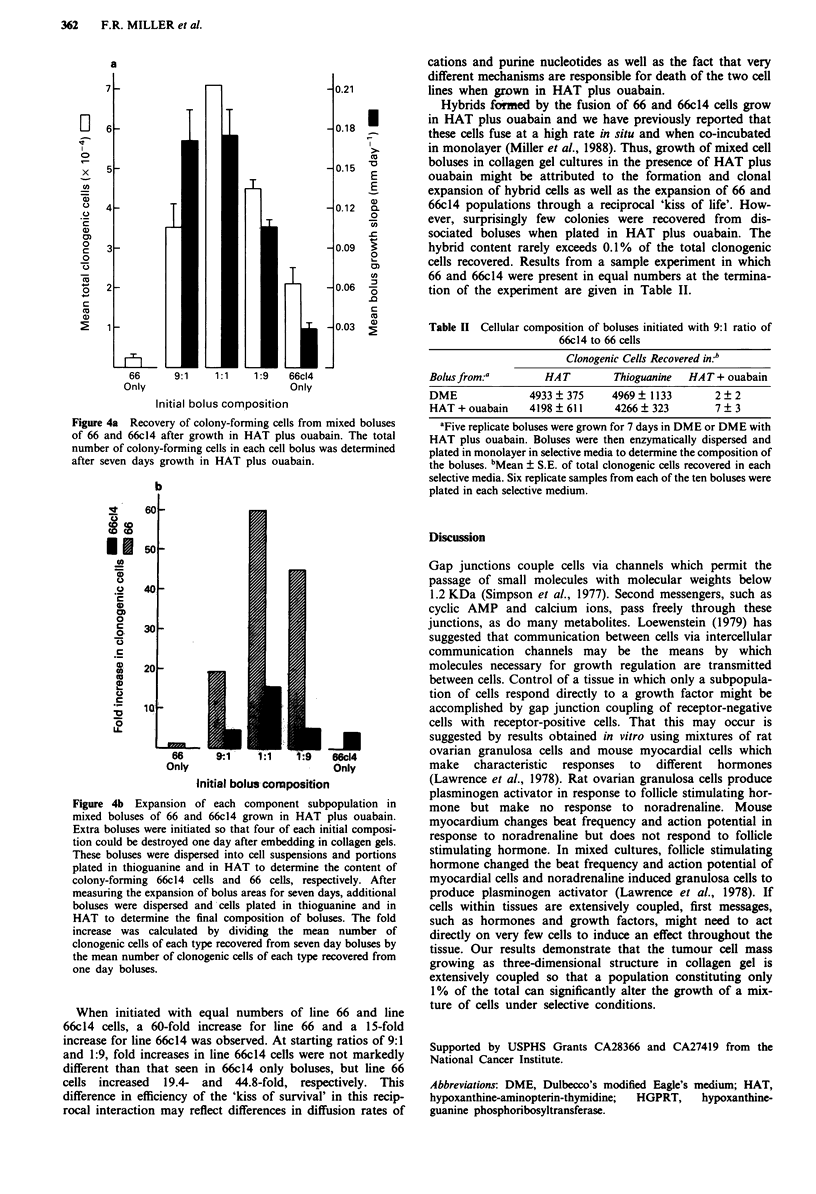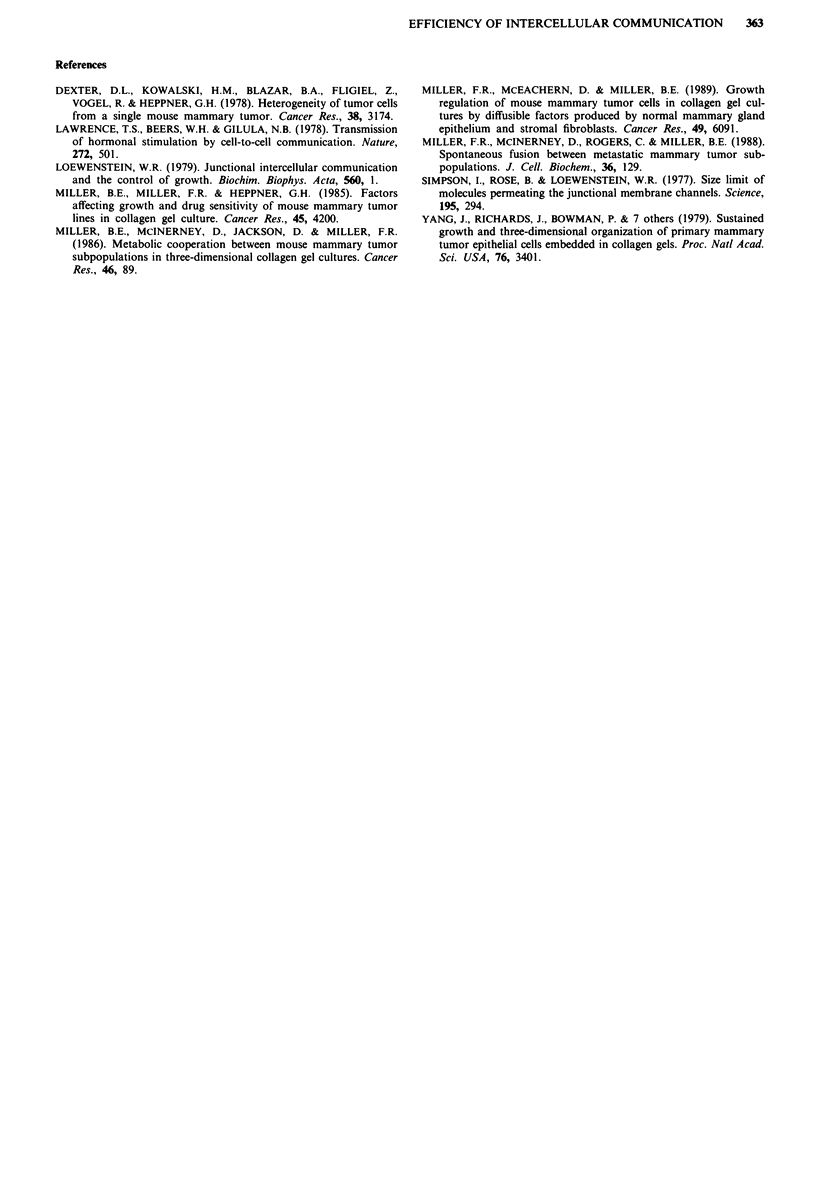# Efficiency of communication between tumour cells in collagen gel cultures

**DOI:** 10.1038/bjc.1990.297

**Published:** 1990-09

**Authors:** F.R. Miller, D. McEachern, B.E. Miller

## Abstract

Mixtures of drug-resistant and drug-sensitive tumour cells growing in 3-dimensional boluses in collagen gel matrix are shown to be effectively coupled so that the response of the mixture is significantly influenced by a subpopulation making up only 1% of the total cells.


Br. J. Cancer (1990), 62, 360-363 © Macmillan Press Ltd., 1990

### Efficiency of communication between tumour cells in collagen gel cultures

F.R. Miller, D. McEachern & B.E. Miller

*E. Walter Albachten Department of Immunology, Michigan Cancer Foundation, 110 East Warren Avenue, Detroit, MI 48201, USA*

**Summary** Mixtures of drug-resistant and drug-sensitive tumour cells growing in 3-dimensional boluses in collagen gel matrix are shown to be effectively coupled so that the response of the mixture is significantly influenced by a subpopulation making up only 1% of the total cells.

We are interested in determining the efficiency with which cells might communicate within neoplastic tissues. We have previously described two methods to measure metabolic co-operation between cells growing in three-dimensional arrays in collagen gel culture (Miller *et al.*, 1986). In one, mixtures of wild type cells and cells which are deficient in the enzyme hypoxanthine guanine phosphoribosyl transferase (HGPRT$^-$) and resistant to ouabain are embedded as boluses of cells in collagen matrix and grown in HAT (hypoxanthine, aminopterin, and thymidine) media with ouabain. In this medium, neither cell can grow unless metabolic co-operation occurs. The HGPRT$^-$ cells cannot grow because of their inability to salvage purines required by the block of *de novo* synthesis by aminopterin; purine nucleotides may pass from the wild type cells to the HGPRT$^-$ cells through gap junctions. The wild type cells cannot grow because ouabain inhibits the active transport of $Na^+$ out of the cell via the $Na^+$:$K^+$ pump; excess $Na^+$ can pass through gap junctions into the HGPRT$^-$ ouabain resistant cells which get rid of the excess $Na^+$. In the second method, HGPRT$^-$ cells are mixed with wild type cells to determine the inhibition of growth of HGPRT$^-$ in medium containing thioguanine. The ability of increasing numbers of wild type cells to inhibit growth of a fixed number of HGPRT$^-$ cells is an indicator of the passage of thioguanine-nucleotides from wild-type cells to HGPRT$^-$ cells. We have attempted to determine the minimum composition of either cell type for detectable communication with these two methods.

### Materials and methods

Tumour cell line 66 was derived from a spontaneously arising mammary tumour from a BALB/cfC3H mouse (Dexter *et al.*, 1978). Line 66c14 is a thioguanine-resistant, ouabain-resistant variant isolated from line 66 after mutagenesis with ethyl methanesulfonate (Miller *et al.*, 1986). Both lines were found to be free of *Mycoplasma* contamination, using DNA fluorochrome stain plus UV microscopy by Bionique Laboratories (Saranac Lake, NY). Ouabain, 6-thioguanine, and concentrated HAT mixture were purchased from Sigma Chemical Co., St. Louis, MO. The final concentrations of selective drugs used were as follows: for HAT medium, hypoxanthine, 100 μM; aminopterin, 0.4 μM; thymidine, 16 μM; for thioguanine medium, 60 μM; and for medium containing ouabain, 3 mM. The medium used was Dulbecco's modified Eagle medium (DME) supplemented with 10% fetal calf serum, 2 mM glutamine, 0.1 mM nonessential amino acids, 100 units $ml^{-1}$ penicillin, and 100 μg $ml^{-1}$ streptomycin.

The collagen gel culture system used is a modification of Yang *et al.* (1979). Collagen stock prepared from rat tail fibres dissolved in dilute acetic acid was diluted with concentrated DME, neutralized with NaOH, and 0.5 ml of this collagen mixture was placed in each well of a 24-well plate and allowed to gel. Cell suspensions at the various ratios were prepared, centrifuged to form a pellet, suspended in collagen mixture, and 1 μl of cell suspension placed on the gel surface. This cell bolus was overlaid with 0.4 ml collagen mixture, then with 0.9 ml supplemented DME containing 2-fold concentrated selective drug. The area of the projected image of a bolus was determined with the aid of a Bioquant digitizer plus Bioquant System IV digitizing morphometry computer program (R and M Biometrics, Inc., Nashville, TN). Culture growth was monitored for 7 days and the growth rate expressed as the slope of the regression line found by plotting the square root of the area of the cell bolus versus the day during the period of linear growth (Miller *et al.*, 1989). Growth rate slopes determined for individual cell boluses were used to determine the statistical significance of differences between experimental groups (4–6 replicates per group) using the Student's t-test. Nuclei numbers present in the cell boluses were determined by digesting the collagen with 0.24 N acetic acid, lysing the cells, and counting with a microscope and haemocytometer. Enumeration of colony-forming cells in boluses was accomplished after digesting collagen gels with 2 mg $ml^{-1}$ collagenase type III (Cooper Biomedical, Malvern, PA) as previously described (Miller *et al.*, 1986). Cell suspensions were counted and portions plated in each selective medium (thioguanine, HAT, or HAT plus ouabain). After incubation at 37°C in 10% $CO_2$ atmosphere for two weeks, colonies were fixed, stained, and counted.

### Results

The growth of 66c14 in boluses in the presence of thioguanine was inhibited by line 66 cells in a dose-dependent fashion (Figure 1). The mean growth slopes were incrementally decreased as line 66 cells were incorporated into the cell boluses in ratios of 66c14 to 66 cells of 100:1, 10:1, 1:1, and 1:5. A significant reduction was obtained when 66 cells made up 9% or more of the initiated cell bolus.

When mixed boluses were embedded in collagen and grown in the presence of HAT and ouabain, growth was observed (Table I). Significant growth occurred when as few as 1% of the cells in the initial bolus were line 66 cells (Figure 2) or as few as 5% were 66c14 cells (Figure 3). Evidence for successful metabolic co-operation resulting in increased survival and growth of cells in mixed boluses was provided by a significant increase in nuclei which could be recovered from boluses after 7 days of growth in HAT plus ouabain as well as in significantly increased growth slopes of the expanding bolus areas.

The composition of the boluses at the end of seven days growth in HAT plus ouabain was determined (Figure 4). By comparing the number of colony-forming 66 cells and colony-forming 66c14 which could be recovered from boluses before and after seven days of growth in HAT plus ouabain, the fold-increase for each cell type was determined.

Correspondence: F.R. Miller
Received 13 February 1990; and in revised form 5 April 1990

**Figure 1a** Growth of mixed boluses in thioguanine. Cell boluses consisting of $5 \times 10^4$ line 66cl4 cells mixed with no other cells (x) or with $5 \times 10^2$ (o), $5 \times 10^3$ (●), $5 \times 10^4$ (Δ), or $2.5 \times 10^5$ (▲) line 66 cells were placed in the centre of wells of 24-well plates in medium containing 60 μM thioguanine. Six replicates of each bolus type were monitored for growth over the seven day period.

**Figure 1b** Growth slopes of mixed boluses in thioguanine. Slope of the growth rate was determined by linear regression analysis for individual boluses. The mean of the slopes ± S.E. for six replicate boluses for each ratio is depicted.

**Table I** Bolus expansion in HAT plus ouabain as percent of growth in non-selective media

| *Initial composition of Cell Bolus* | *% of Control Growth in DME*[a] *(mean ± S.E. of 4–8 experiments)* |
|---|---|
| all 66cl4 | 8.1 ± 4.1 |
| 9:1, 66cl4:66 | 61.5 ± 9.8 |
| 1:1, 66cl4:66 | 50.1 ± 4.3 |
| 1:9, 66cl4:66 | 27.3 ± 7.3 |
| all 66 | 0.1 ± 0.1 |

[a]Comparison of growth slopes.

**Figure 2** Efficiency of communication in HAT plus ouabain with line 66 in excess. Slopes were determined by linear regression analysis of measurements over seven days for six replicates of each composition: $5 \times 10^4$ line 66 cells only: $4.75 \times 10^4$ line 66 plus $2.5 \times 10^3$ line 66cl4; $4.5 \times 10^4$ line 66 cells plus $5 \times 10^3$ line 66cl4 and $3.75 \times 10^4$ line 66 plus $1.25 \times 10^4$ line 66cl4.

**Figure 3** Efficiency of communication in HAT plus ouabain with line 66cl4 in excess. Slopes were determined by linear regression analysis of measurements over seven days for six replicates of each composition: $5 \times 10^4$ line 66cl4 cells only; $4.95 \times 10^4$ line 66cl4 plus $5 \times 10^2$ line 66 cells; $4.5 \times 10^4$ line 66cl4 plus $5 \times 10^3$ line 66; and $3.75 \times 10^4$ line 66cl4 plus $1.25 \times 10^4$ line 66.

Boluses containing only line 66 cells did not expand in HAT plus ouabain and there was no net increase in clonogenic cells over the seven day period. Boluses containing only line 66cl4 cells expanded slowly in HAT plus ouabain and recovery of clonogenic cells increased 4.1-fold over the 7-day period. This is consistent with our previous report that mouse mammary tumour cells are quite resistant to methotrexate in collagen gel cultures (Miller *et al.*, 1985). Thus, concentrations of aminopterin in HAT media which completely inhibits HGPRT⁻ cells in monolayer culture were insufficient to inhibit completely growth of HGPRT⁻ cells growing as boluses in collagen gel cultures. That growth of HGPRT⁻ cells was inhibited by HAT in collagen gel cultures is shown by Table I; line 66cl4 growth was inhibited by more than 90%.

**Figure 4a** Recovery of colony-forming cells from mixed boluses of 66 and 66cl4 after growth in HAT plus ouabain. The total number of colony-forming cells in each cell bolus was determined after seven days growth in HAT plus ouabain.

**Figure 4b** Expansion of each component subpopulation in mixed boluses of 66 and 66cl4 grown in HAT plus ouabain. Extra boluses were initiated so that four of each initial composition could be destroyed one day after embedding in collagen gels. These boluses were dispersed into cell suspensions and portions plated in thioguanine and in HAT to determine the content of colony-forming 66cl4 cells and 66 cells, respectively. After measuring the expansion of bolus areas for seven days, additional boluses were dispersed and cells plated in thioguanine and in HAT to determine the final composition of boluses. The fold increase was calculated by dividing the mean number of clonogenic cells of each type recovered from seven day boluses by the mean number of clonogenic cells of each type recovered from one day boluses.

When initiated with equal numbers of line 66 and line 66cl4 cells, a 60-fold increase for line 66 and a 15-fold increase for line 66cl4 was observed. At starting ratios of 9:1 and 1:9, fold increases in line 66cl4 cells were not markedly different than that seen in 66cl4 only boluses, but line 66 cells increased 19.4- and 44.8-fold, respectively. This difference in efficiency of the 'kiss of survival' in this reciprocal interaction may reflect differences in diffusion rates of cations and purine nucleotides as well as the fact that very different mechanisms are responsible for death of the two cell lines when grown in HAT plus ouabain.

Hybrids formed by the fusion of 66 and 66cl4 cells grow in HAT plus ouabain and we have previously reported that these cells fuse at a high rate *in situ* and when co-incubated in monolayer (Miller *et al.*, 1988). Thus, growth of mixed cell boluses in collagen gel cultures in the presence of HAT plus ouabain might be attributed to the formation and clonal expansion of hybrid cells as well as the expansion of 66 and 66cl4 populations through a reciprocal 'kiss of life'. However, surprisingly few colonies were recovered from dissociated boluses when plated in HAT plus ouabain. The hybrid content rarely exceeds 0.1% of the total clonogenic cells recovered. Results from a sample experiment in which 66 and 66cl4 were present in equal numbers at the termination of the experiment are given in Table II.

**Table II** Cellular composition of boluses initiated with 9:1 ratio of 66cl4 to 66 cells

| | Clonogenic Cells Recovered in:[b] | | |
|---|---|---|---|
| *Bolus from:*[a] | *HAT* | *Thioguanine* | *HAT* + ouabain |
| DME | 4933 ± 375 | 4969 ± 1133 | 2 ± 2 |
| HAT + ouabain | 4198 ± 611 | 4266 ± 323 | 7 ± 3 |

[a]Five replicate boluses were grown for 7 days in DME or DME with HAT plus ouabain. Boluses were then enzymatically dispersed and plated in monolayer in selective media to determine the composition of the boluses. [b]Mean ± S.E. of total clonogenic cells recovered in each selective media. Six replicate samples from each of the ten boluses were plated in each selective medium.

### Discussion

Gap junctions couple cells via channels which permit the passage of small molecules with molecular weights below 1.2 KDa (Simpson *et al.*, 1977). Second messengers, such as cyclic AMP and calcium ions, pass freely through these junctions, as do many metabolites. Loewenstein (1979) has suggested that communication between cells via intercellular communication channels may be the means by which molecules necessary for growth regulation are transmitted between cells. Control of a tissue in which only a subpopulation of cells respond directly to a growth factor might be accomplished by gap junction coupling of receptor-negative cells with receptor-positive cells. That this may occur is suggested by results obtained *in vitro* using mixtures of rat ovarian granulosa cells and mouse myocardial cells which make characteristic responses to different hormones (Lawrence *et al.*, 1978). Rat ovarian granulosa cells produce plasminogen activator in response to follicle stimulating hormone but make no response to noradrenaline. Mouse myocardium changes beat frequency and action potential in response to noradrenaline but does not respond to follicle stimulating hormone. In mixed cultures, follicle stimulating hormone changed the beat frequency and action potential of myocardial cells and noradrenaline induced granulosa cells to produce plasminogen activator (Lawrence *et al.*, 1978). If cells within tissues are extensively coupled, first messages, such as hormones and growth factors, might need to act directly on very few cells to induce an effect throughout the tissue. Our results demonstrate that the tumour cell mass growing as three-dimensional structure in collagen gel is extensively coupled so that a population constituting only 1% of the total can significantly alter the growth of a mixture of cells under selective conditions.

Supported by USPHS Grants CA28366 and CA27419 from the National Cancer Institute.

*Abbreviations*: DME, Dulbecco's modified Eagle's medium; HAT, hypoxanthine-aminopterin-thymidine; HGPRT, hypoxanthine-guanine phosphoribosyltransferase.

**References**

DEXTER, D.L., KOWALSKI, H.M., BLAZAR, B.A., FLIGIEL, Z., VOGEL, R. & HEPPNER, G.H. (1978). Heterogeneity of tumor cells from a single mouse mammary tumor. *Cancer Res.*, **38**, 3174.

LAWRENCE, T.S., BEERS, W.H. & GILULA, N.B. (1978). Transmission of hormonal stimulation by cell-to-cell communication. *Nature*, **272**, 501.

LOEWENSTEIN, W.R. (1979). Junctional intercellular communication and the control of growth. *Biochim. Biophys. Acta*, **560**, 1.

MILLER, B.E., MILLER, F.R. & HEPPNER, G.H. (1985). Factors affecting growth and drug sensitivity of mouse mammary tumor lines in collagen gel culture. *Cancer Res.*, **45**, 4200.

MILLER, B.E., MCINERNEY, D., JACKSON, D. & MILLER, F.R. (1986). Metabolic cooperation between mouse mammary tumor subpopulations in three-dimensional collagen gel cultures. *Cancer Res.*, **46**, 89.

MILLER, F.R., MCEACHERN, D. & MILLER, B.E. (1989). Growth regulation of mouse mammary tumor cells in collagen gel cultures by diffusible factors produced by normal mammary gland epithelium and stromal fibroblasts. *Cancer Res.*, **49**, 6091.

MILLER, F.R., MCINERNEY, D., ROGERS, C. & MILLER, B.E. (1988). Spontaneous fusion between metastatic mammary tumor subpopulations. *J. Cell. Biochem.*, **36**, 129.

SIMPSON, I., ROSE, B. & LOEWENSTEIN, W.R. (1977). Size limit of molecules permeating the junctional membrane channels. *Science*, **195**, 294.

YANG, J., RICHARDS, J., BOWMAN, P. & 7 others (1979). Sustained growth and three-dimensional organization of primary mammary tumor epithelial cells embedded in collagen gels. *Proc. Natl Acad. Sci. USA*, **76**, 3401.